# Supplementary material for: Limited Cheese Intake Paradigm Replaces Patterns of Behavioral Disorders in Experimental PTSD: Focus on Resveratrol Supplementation
Source: Int J Mol Sci. 2023 Sep 20;24(18):14343. doi: 10.3390/ijms241814343 (PMC10532287; doi:10.3390/ijms241814343)
Supplement: Supplementary file 1 [file ijms-24-14343-s001.zip › ijms-2582307-supplementary.pdf]

**Supplementary Materials**

**S1. Obtaining control semi-hard cheese**

For the production of semi-hard cheese, raw cow's milk was used, its composition is presented in Table S1.

**Table S1.** Composition of milk.

| Fat | Protein | dry skimmed milk residue | Lactose | Urea |
|---|---|---|---|---|
| 3.18 | 2.67 | 7.98 | 4.63 | 15.1 |

First, 5 liters of milk were poured into the TREMAS-MIX 3.0 cheese maker. At the beginning of the process, the pH of the milk was recorded 6.03 using a pH meter. Next, the milk was pasteurized at a temperature of 65°C for 15 minutes. After that, the pasteurized milk was cooled to a temperature of 42 ° C, and 0.1 g/l of calcium chloride was added to it, which can dissolve well in milk at this temperature. Then the mixture was cooled to a temperature of 36 °C, this temperature of 36 °C is optimal for the subsequent fermentation of the mixture by adding starter (Choozit), keeping the mixture for 20–30 minutes, adding 0.01 g/l of rennet (Microbial Meito Rennet).

As a result of fermentation, the mixture coagulates with the formation of a clot. After curdling, the first cutting of the clot was carried out, after a few minutes a second cutting was carried out until the desired grain size was obtained. The cut clot was mixed, ensuring the setting of the cheese grain. Next, the mixture was heated to a temperature of 40°C, and held for 30 min.

Next, the whey was drained, cheese grains were placed in a mold and pressed for 3 hours. Finally, the cheese was salted using a 20% saline solution for 2 hours, left to dry overnight at 9°C, and covered by food-grade latex. The cheese was kept for 30 days for ripening at 9-13°C.

**S2. Obtaining semi-hard cheese fortified with encapsulated resveratrol**

For fortification, 130 mg of resveratrol were added per liter of milk. Encapsulation of resveratrol in liposomes was prepared using lipid components of phosphatidylcholine, palmitic acid choline ester and sugar. Liposomes were obtained by a modified method. 650 mg of resveratrol was dissolved in 20 ml of ethanol with stirring on a magnetic stirrer for 10 minutes. Phosphatidylcholine, 250 mg, and cholesterol, 40 mg, were then weighed and added to the astaxanthin solution. Transferred to a round bottom flask for evaporation. The evaporation was carried out on a rotary evaporator and starts without heating the water bath. The resulting film was dried in a desiccator. Next, hydration was carried out with 25 ml of 1% sucrose solution with gentle stirring and heating in a water bath.

The result was a brightly colorless liposomal suspension, quite homogeneous. Next, the resulting liposomal suspension was added to the prepared milk (500 ml) and transferred to the rest of the milk in the cheese maker. Further, everything is in accordance with the method of obtaining control cheese. Thus, a good clot was obtained.

**S3. HPLC method for the determination of resveratrol in semi-hard cheese fortified with encapsulated resveratrol**

For analysis, a sample weighing about 0.5 g was placed in centrifugation tubes with a capacity of 15.0 ml, and 5.0 ml of a mixture of acetonitrile - 1% formic acid in water in a ratio of 80: 20 was added with a pipette. The tubes were mixed for 30 minutes for Vortex Labtex (China). Then the tubes were centrifuged for 15 min at 7500 rpm. / min. and a temperature of 0 °C in a Thermo ST16R centrifuge (Germany). The supernatant was transferred into tubes and analyzed.

Quantitative determination of resveratrol in products by HPLC-UV was carried out on an Agilent 1260 Infinity II liquid chromatograph (USA) with a diode array UV detector.

Chromatographic separation was performed on an Agilent Poroshell 120 EC-C18 (3.0 mm × 100 mm × 2.7 μm) reverse phase column (p/n 695975-302) additionally protected by a Poroshell 120 EC-C18 guard column (3.0 mm × 5 mm × 2.7 μm) manufactured by Agilent (p/n 823750-911). A mixture of 1% solution of acetic acid in water (eluent A) and acetonitrile (eluent B) was used as a mobile phase in isocratic mode at component ratios (A:B) of 78:22.

The flow rate of the mobile phase was 0.7 ml/min. The column oven temperature was set to 30°C. Samples were injected in a volume of 2 μl. Detection was carried out at a wavelength of 304 nm with a slit width of 4 nm.

fat soluble vitamins. Buffer: Water and ACN:MeOH 75:25.

Gradients: after 0min, 1min, 26min, 26.1min % of the second by 90, 100, 100, 90% respectively. At 45°C, volume 1.5ml/min (0.9ml/min), and 325nm (7, 14, 20min at 210, 270, 450nm respectively). Sample 5 μl and stop at 26.1 pos time 2.

The identification of components was carried out using working standard samples by comparing the retention times and UV spectra of the analytes. Quantitative determination was carried out by the external standard method using a calibration curve.

**S4. Dynamics of changes in the content of resveratrol in fortified semi-hard cheese during ripening period**

HPLC analysis was performed to determine the stability of resveratrol during cheese ripening and yogurt storage. A typical chromatogram of resveratrol in cheese extract is shown in Figure S1.

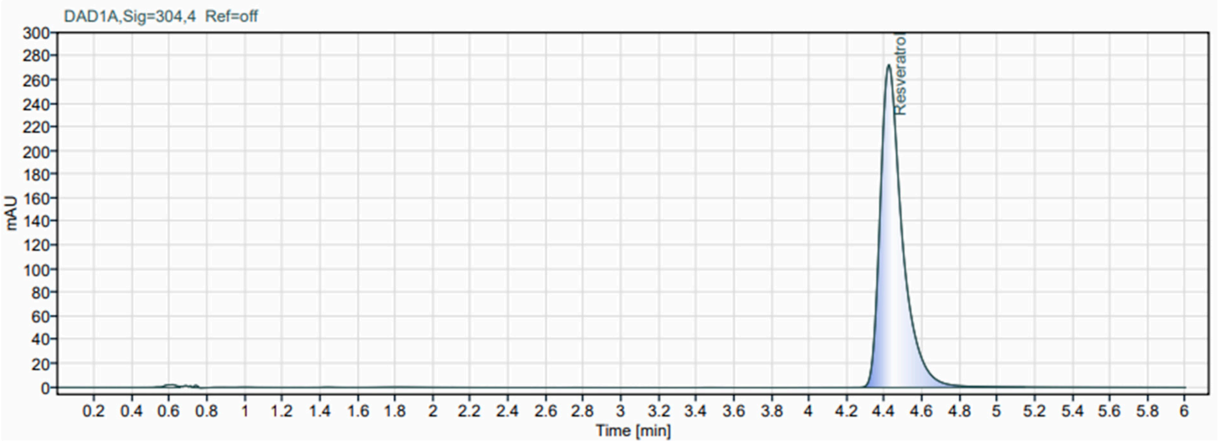


**Figure S1.** Chromatogram of resveratrol added to cheese in the form of liposomes.

The chromatographic peak of resveratrol in the sample appears at a retention time of 4.42 minutes.

**Table S2.** The content of resveratrol in traditional and encapsulated form during cheese ripening

| | Mean value ± standard deviation, (mg/g) | | | |
|---|---|---|---|---|
| | First week | Second week | Third week | Fourth week |
| Resveratrol content | 0.98 ± 0.04 | 0.86 ± 0.10 | 0.87 ± 0.11 | 1.03 ± 0.13 |

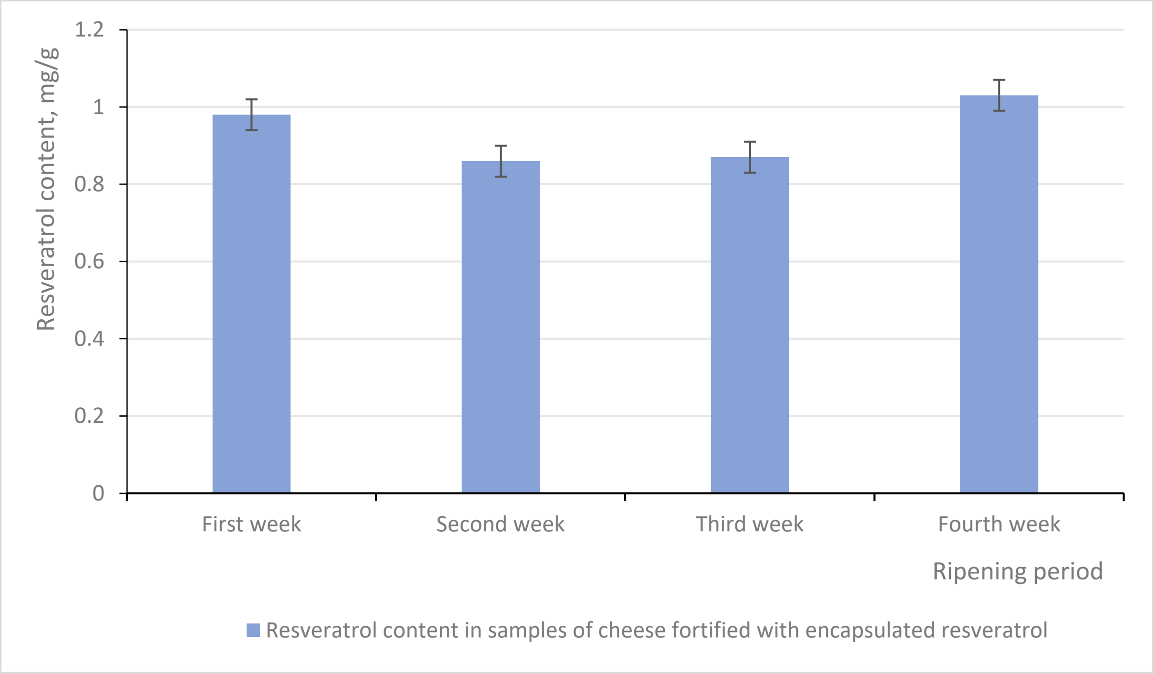


**Figure S2.** The content of resveratrol in traditional and encapsulated form during cheese ripening.

The graph demonstrates the stability of the liposome ingredient resveratrol in semi-hard cheese throughout the ripening period. This proves the feasibility of encapsulating resveratrol for its protection and better preservation in the product.
